# Characteristics of Sleep Structure Assessed by Objective Measurements in Patients With Amnestic Mild Cognitive Impairment: A Meta-Analysis

**DOI:** 10.3389/fneur.2020.577126

**Published:** 2020-11-12

**Authors:** Sijie Cai, Tingting Li, Li Zhang, Longhua Shi, Jingling Liao, Wenfang Li, Guangwen Cheng, Wei Tan, Shuang Rong

**Affiliations:** ^1^Department of Nutrition and Food Hygiene, School of Public Health, Medical College, Wuhan University of Science and Technology, Wuhan, China; ^2^Hubei Province Key Laboratory of Occupational Hazard Identification and Control, Wuhan University of Science and Technology, Wuhan, China; ^3^Department of Neurology, Hubei Provincial Hospital of Integrated Chinese & Western Medicine, Wuhan, China; ^4^Community Health Service Center of Qingling, Wuhan, China; ^5^Hospital of Wuhan University of Science and Technology, Wuhan, China

**Keywords:** amnestic mild cognitive impairment, sleep structure, polysomnography, actigraphy, meta-analysis

## Abstract

**Objectives:** This study aims to explore the differences of sleep structure between patients with amnestic mild cognitive impairment (aMCI) and elderly people with normal cognition, which will help to provide evidence for the relationship between sleep disturbances and cognitive impairment.

**Methods:** A systematic review and meta-analysis were conducted on the literature on sleep parameters obtained by polysomnography or actigraphy in patients with aMCI. The PubMed and EMBASE databases were searched up to April 2020. Inclusion and exclusion criteria were established according to evidence-based medicine methods, and data of all eligible studies were meta-analyzed using the Review Manager 5.3 software.

**Results:** Among the 1,171 literature articles on sleep structure of patients with MCI, eight case-control studies met the inclusion criteria and were included in this meta-analysis. A total of 278 subjects were included, of which 103 were patients with aMCI and 175 were elderly people with normal cognition. The results showed that sleep efficiency (SE) and slow wave sleep (SWS) of patients with aMCI were significantly lower than those of healthy elderly people. Compared with the control group, the percentage of stage 1 of non-rapid eye movement (N1%) in the aMCI patients group increased, and the percentage of stage 2 of non-rapid eye movement (N2%) decreased.

**Conclusions:** Patients with aMCI may experience more severe sleep disturbances than normal cognitive elderly people. There were specific changes, especially in SE and SWS, in the sleep structure of patients with aMCI when compared to those with normal cognition.

## Introduction

Around the world, there will be one new case of dementia every 3 seconds (1). In 2019, it was estimated that there were over 50 million people living with dementia globally, and this number is said to more than triple to 152 million by 2050 (2). Mild cognitive impairment (MCI) is the stage between the expected cognition decline of normal aging and the more serious decline of dementia (3). Between 10 and 20% of people older than 65 years were diagnosed with MCI in 2015 (4), and the rate of MCI patients who will go on to develop dementia is about 14.9% within 2 years (5). Not all forms of MCI evolve into Alzheimer's disease (AD). Amnestic MCI (aMCI) refers to impairment purely in one's ability to recall stored information, which is the most common type of MCI and has a higher risk of developing AD (6). In view of the lack of effective treatment for AD (7), early detection and prevention measures taken in the aMCI stage are of great importance.

Over the last decade, sleep disturbance has received mounting interest as another important risk factor for cognitive decline. Individuals with sleep problems had a 1.55 (95%CI: 1.25–1.93) and 1.65 (95%CI: 1.45–1.86) times higher risk of AD and cognitive impairment than individuals without sleep problems, respectively (8). Approximately 15% of AD in the population may be attributed to sleep problems (8). Meanwhile, sleep disorder is considered as a concurrent symptom of cognitive impairment, a review found that 60~70% of people with cognitive impairment or dementia have sleep disturbances (9). And in turn a drop in sleep quality also worsens cognitive impairment, especially the deterioration of memory, because the memory consolidation process is completed during sleep (10, 11). There may be a bidirectional relationship between sleep disturbance and cognitive impairment, which has been left unlinked.

Sleep disturbances can be evaluated by subjective and objective methods (12). Commonly used subjective assessment methods for sleep quality include the Pittsburgh Sleep Quality Index (PSQI) (13), Epworth Sleepiness Scale (ESS) (14, 15), and a sleep diary. In terms of objective methods for monitoring sleep state, polysomnography (PSG) and actigraphy are highly correlated with sleep state (12, 16). PSG is the agreed method for evaluating night time sleep duration and fragmentation and to differentiate sleep staging (17). PSG uses various methods to simultaneously and continuously record neurophysiologic, cardiopulmonary, and other physiologic parameters during an entire night. Actigraphy is quantitative but relies on rest-activity rhythms as a surrogate for sleep-wake activity.

The relationship between sleep structure evaluated by an objective method and aMCI is unclear. Some studies explored the changes of the sleep structure in patients with aMCI, but the conclusions were not consistent, especially in total sleep time (TST), slow wave sleep (SWS), and wake after sleep onset (WASO) (18, 19). Our study aimed to clarify the characteristic change of objective sleep structure in patients with aMCI by conducting a meta-analysis. We synthesized study results from sleep state objectively detected by the PSG and actigraphy, and attempted to clarify the differences of sleep structure between aMCI patients and normal cognitive elderly people.

## Methods

### Inclusion Criteria

(1) Original articles from observational studies (cross-sectional, case-control, and retrospective/prospective cohort) were included in this review; (2) there were clear criteria for the selection and diagnosis of participants. Patients should be diagnosed with aMCI, and the control group must contain healthy elderly people with normal cognition; (3) the detection methods of the outcomes were clear. The method for evaluating sleep quality should contain at least one objective measure, PSG or actigraphy; (4) the outcomes contained sleep structure and sleep parameters; (5) sample size, mean, and standard deviation were provided.

### Exclusion Criteria

(1) The study was a duplicate report; (2) the research design was defective and of poor quality; (3) the outcomes were incomplete or unclear, and quantitative data cannot provide the mean and standard deviation; (4) the method of statistics was wrong and could not be corrected.

### Data Sources and Search Strategy

We collected eligible studies that evaluated sleep disturbances in patients with aMCI compared to cognitively normal controls, and the measurement for monitoring sleep structure were required to contain PSG and/or actigraphy as the objective evidence. We conducted a systematic literature search using the PubMed and EMBASE databases, limited to articles published in English. The databases were searched up to April 2020. We also reviewed the references of the related studies to explore more potential candidates. Take the PubMed query as an example, the search terms included (“mild cognitive impairment[Title/Abstract]” OR “amnestic mild cognitive impairment[Title/Abstract]” OR “cognitive decline[Title/Abstract]” OR “cognitive dysfunction[Title/Abstract]”) AND (“sleep structure[Title/Abstract]” OR “sleep architecture[Title/Abstract]” OR “sleep parameter[Title/Abstract]” OR “sleep disturbance[Title/Abstract]” OR “sleep disorder[Title/Abstract]” OR “sleep wake disorder[Title/Abstract]”).

### Review Selection and Data Extraction

In accordance with the formulated inclusion and exclusion criteria according to evidence-based medicine, firstly the titles were reviewed for obvious exclusions. Then we reviewed abstracts for further exclusions. If it was unclear whether the article should be excluded after the abstract was read, we would review the full text. Two reviewers (Cai SJ and Li TT) selected articles and extracted data independently. Finally, their lists of articles were compared to ensure that the same studies had been included. The two reviewers resolved the discrepancies by discussion. If the discrepancies could not be solved, the final decision was made by a third person (Rong S).

### Literature Quality Assessment

The tool used for evaluating the quality of the studies was the Newcastle-Ottawa Scale (NOS), NOS is commonly used to evaluate the quality of case-control and cohort studies (20). Our study included eight case-control studies. Two reviewers (Cai SJ and Li TT) evaluated the included studies independently and they reached a consensus by discussion. The criteria of the NOS scale includes three categories of selection (four items, four stars), comparability (one item, two stars), and exposure (three items, three stars) for a total of nine stars. A total score of six stars or above was considered of high quality.

### Data Synthesis and Statistical Analysis

RevMan 5.3 software was used in our meta-analysis. The standard mean difference (SMD) with 95%CI was applied. To begin, heterogeneity was assessed using *I*^2^ statistics. An *I*^2^ value of 75% and above indicates a high degree of heterogeneity (21). The fixed-effect model was used if the result of the heterogeneity test showed *I*^2^ <50%. Otherwise, heterogeneity was considered to exist in the included studies, and the random effect model would be used for the combined analysis. Subgroup analysis was used to analyze the sources of heterogeneity. A funnel plot was drawn to judge publication bias. The SMD as the x-axis and the SE (SMD) as the y-axis were used to examine publication bias. A *p* <0.05 was considered statistically significant.

## Results

### Search Results

**Figure 1:**
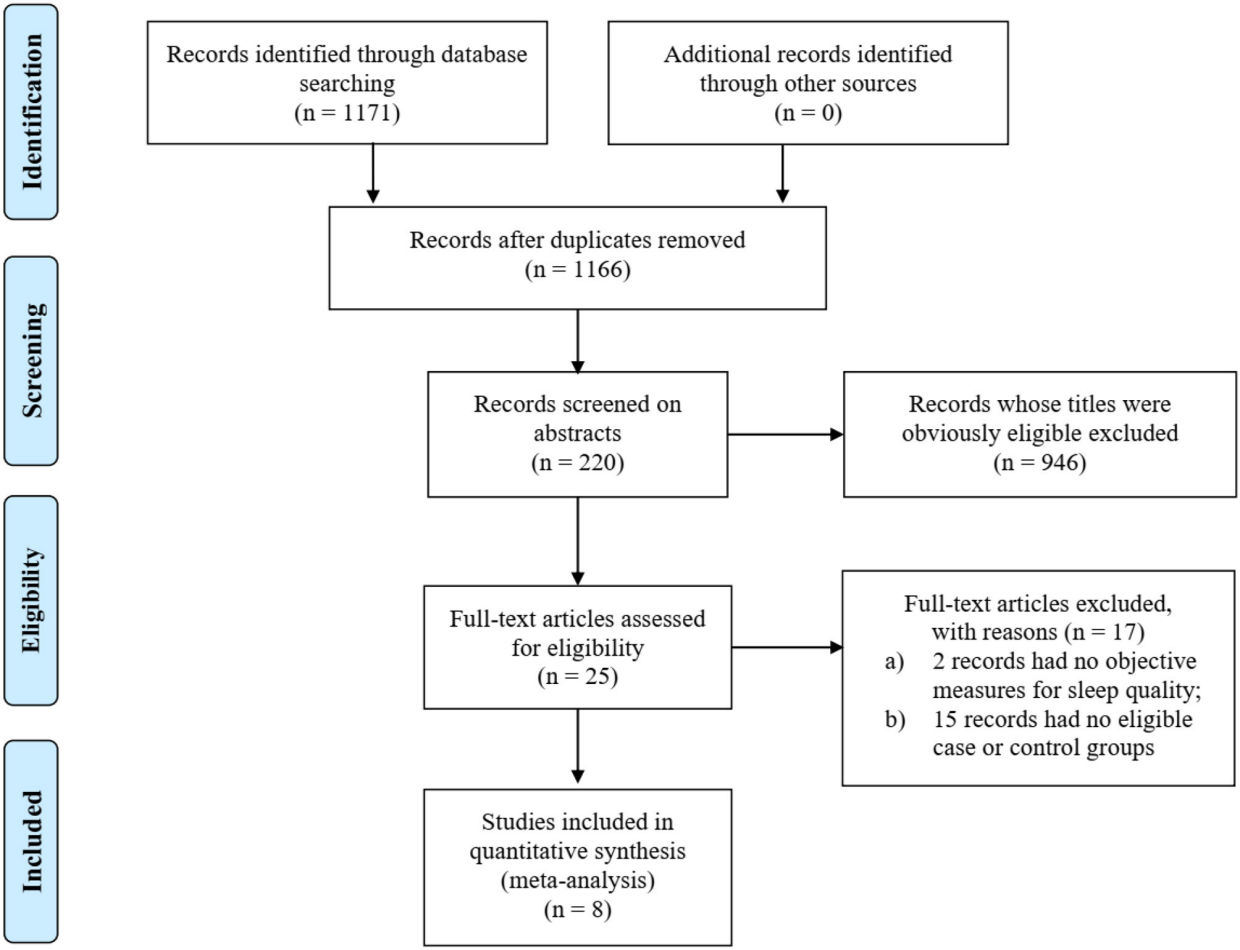
PRISMA flowchart of searching and selection guidelines.

By searching PubMed and EMBASE, we found 1,171 relevant studies. After excluding 951 studies that did not meet the inclusion criteria, abstracts of 220 studies were screened. Then, the full text of the 25 potentially eligible articles were read. Finally, eight case-control studies fulfilled the eligibility criteria and were included in this meta-analysis. The literature search method was conducted as shown in Figure 1.

### Quality Assessment of Included Studies

Quality assessment was conducted based on the criteria of NOS. All the articles scored more than six stars in terms of NOS, so all the articles were considered of high quality (Table 1).

**Table 1:** Results of NOS quality assessment of the eight included studies.

**Studies**	**Selection**				**Comparability**	**Exposure**			**Total**
	**Adequate definition of cases**	**Representativeness of the cases**	**Selection of controls**	**Definition of controls**	**Control for important factor**	**Ascertainment of exposure**	**Same methods of ascertainment for cases and controls**	**Non-response rate**	
Gorgoni et al. (22)	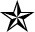	–	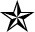	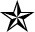	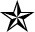 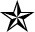	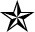	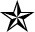	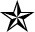	8
Hayes et al. (23)	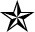	–	–	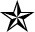	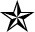 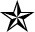	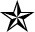	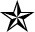	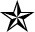	7
Brayet et al. (23)	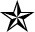	–	–	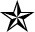	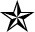 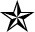	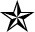	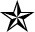	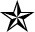	7
Reda et al. (24)	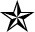	–	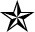	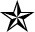	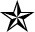 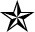	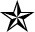	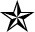	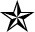	8
Terpening et al. (25)	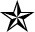	–	–	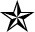	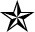 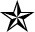	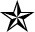	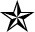	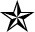	7
Westerberg et al. (26)	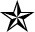	–	–	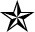	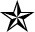 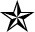	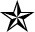	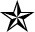	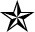	7
Westerberg et al. (27)	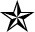	–	–	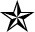	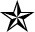 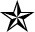	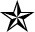	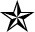	–	6
Wams et al. (28)	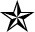	–	–	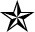	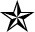 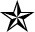	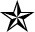	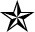	–	6

*represents the score for the NOS scale.

### Baseline Characteristics of Studies and Samples

Information of the eight included studies (22–29), such as publication time, countries, major criteria of aMCI, and characteristics of the aMCI and control samples are listed (Table 2).

**Table 2:** Characteristics and information of the eight studies and samples.

**Studies**	**Country/regions**	**Major criteria for aMCI**	**Gender**		**Age**		**Measure for sleep quality**
			**Case (M/F)**	**Control (M/F)**	**Case**	**Control**	
Gorgoni et al. (22)	Italy	(1) MMSE; (2) DSM-IV criteria; (3) brain neuroimaging(MRI or CT); (4) RAVLT; (5) ADL/IADL	6/9	10/5	71.10 (2.28)	70.80 (1.63)	PSG
Hayes et al. (23)	US	(1) Mini-mental state examination score of more than 23; (2) clinical dementia rating scale score ≤ 0.5; (3) score <5 on the short version of the geriatric depression scale	1/5	3/26	84.8 (6.6)	87.5 (4.0)	In-home actigraphy sensors; the sleep disturbance symptom questionnaire
Braye et al. (23)	Canada	(1) A performance rated more than or equal to 1.5 standard deviation below the standardized mean on at least two tests (or variables) in one cognitive domain; (2) cognitive decline not better explained by a medical or psychiatric condition or by substance abuse; (3) preserved activities of daily living of past and present capacity; (4) significant impairment in memory with or without impairment in other cognitive domains	4/18	10/22	63.9 (7.7)	63.7 (6.6)	PSG; quantitative EEG
Reda et al. (24)	Italy	(1) MMSE; (2) state trait anxiety index; (3) HDRS; (4) brain neuroimaging (MRI or CT); (5) RAVLT; (6) ADL/IADL	8/12	12/8	72.20 (1.79)	70.35 (1.4)	PSG; PSQI; ESS; KSD
Terpening et al. (25)	Australia	(1) MMSE; (2) structured clinical interview for DSM-IV-R; (3) TMT-A; (4) the logical memory subtest of the Wechsler memory scale—III; (5) language assess; (6) TMT-B	8/6	19/21	72.6 (8.1)	63.5 (8.9)	PSG; Horne-Ostberg morningness-eveningness questionnaire; PSQI; ESS; actigraphy; sleep diaries
Westerberg et al. (26)	US	(1) Scores in one or more cognitive domains, including at least one declarative memory measure, were ≥ 1.5 standard deviations (SD) below the mean for individuals of comparable gender, age, and education level; (2) not impaired in daily living activities and did not meet clinical criteria for dementia	2/8	3/7	71.1	72.5	Wrist-worn activity sensor; PSQI; ESS; KSD
Westerberg et al. (27)	US	(1) Scores in one or more cognitive domains, including at least one declarative memory measure, were ≥ 1.5 standard deviations(SD) below the mean for individuals of comparable gender, age, and education level; (2) not impaired in daily living activities and did not meet clinical criteria for dementia	1/7	3/11	75.6 (7.2)	72.7 (5.1)	PSG; wrist-worn actigraphy; sleep diary
Wams et al. (28)	UK	(1) MMSE; (2) Petersen's criteria 1999	4/4	7/6	77.1 (4.0)	73.8 (4.6)	Wrist-worn actigraphy; a semi-standardized diary; Jupiter sleep questionnaire; PSQI

### Data Analysis of Sleep Parameters

#### TST

**Figure 2:**
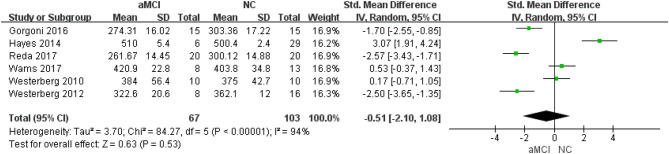
Meta-analysis of TST between the aMCI and NC groups.

Six (22, 24, 26–29) included studies showed that there was no significant difference between the aMCI and NC groups (SMD = −0.51, 95%CI (−2.10~1.08), *P* = 0.53) (Figure 2).

##### Subgroup analysis of TST

**Figure 3:**
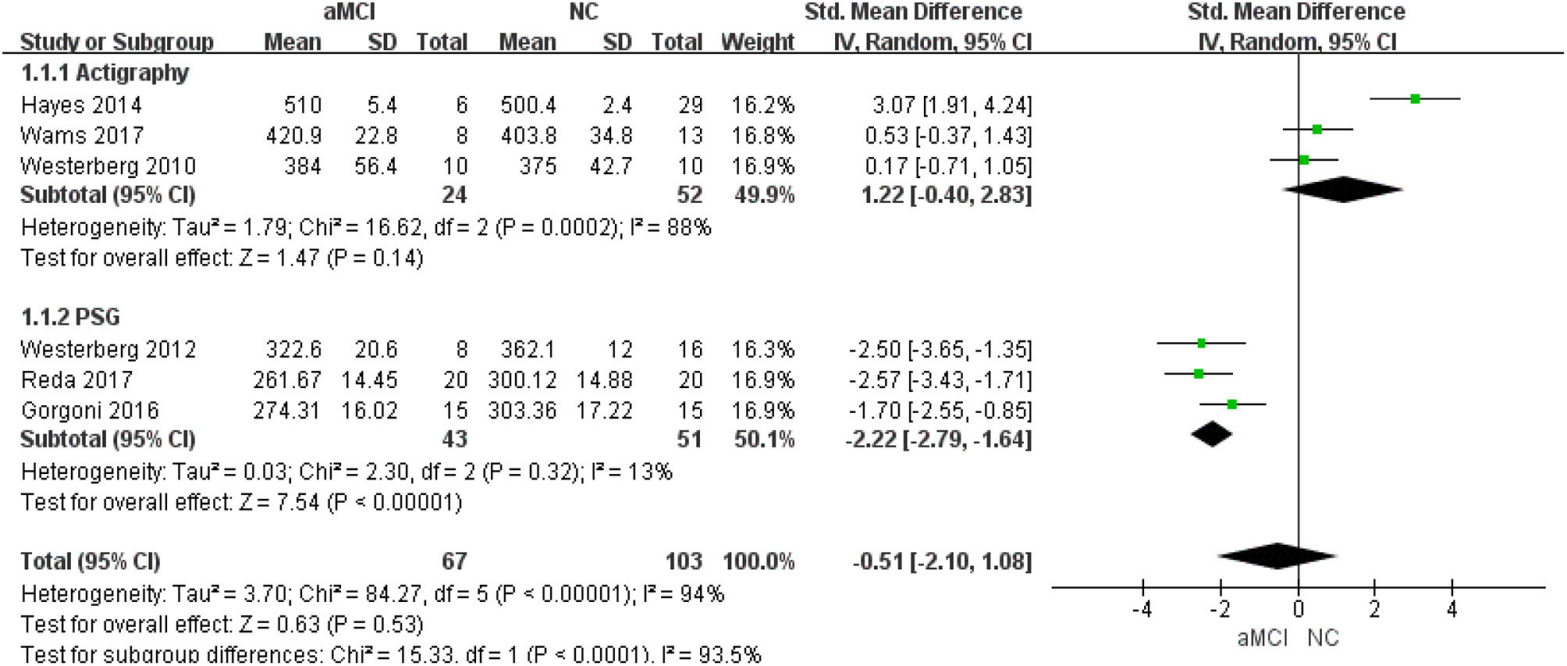
Subgroup analysis of different measurements in relation to TST.

Different measurements: three (22, 24, 27) studies provided detailed PSG data, while three (26, 28, 29) provided actigraphy data. In the actigraphy subgroup, no significant difference was observed between the aMCI and NC groups [SMD = 1.22, 95%CI (−0.40~2.83), *P* = 0.14]. In the PSG subgroup, shorter TST was observed in the aMCI groups [SMD = −2.22, 95%CI (−2.79~-1.64), *P* < 0.00001] (Figure 3).

#### Sleep Efficiency (SE)

**Figure 4:**
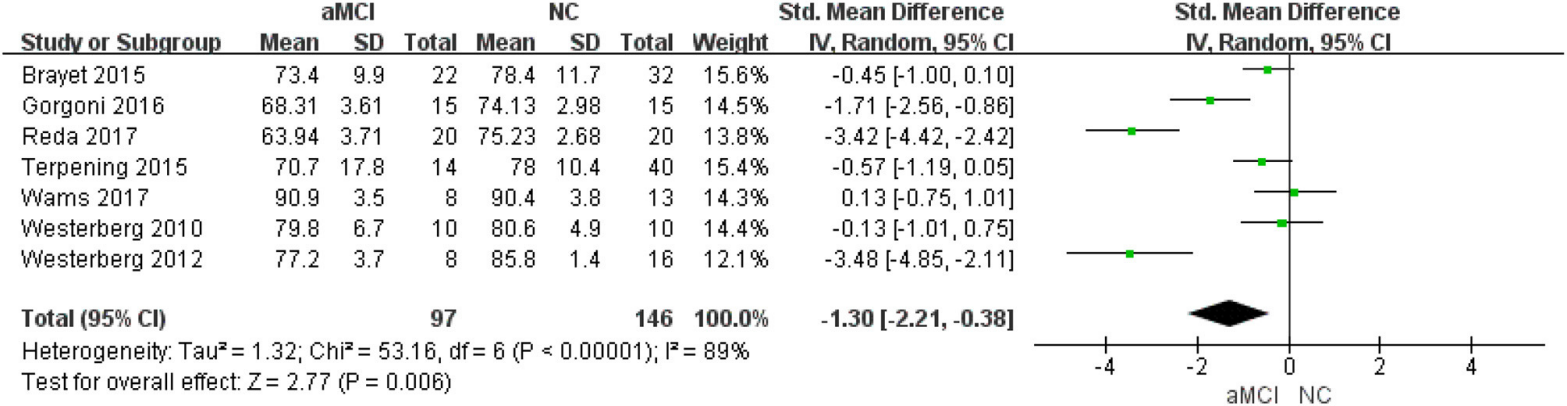
Meta-analysis of SE between the aMCI and NC groups.

Seven (22–28) included studies showed that the NC group had better SE [SMD = −1.30, 95%CI (−2.21~-0.38), *P* =0.006] (Figure 4).

##### Subgroup analysis of SE

Different measurements: five (22–25, 27) studies provided detailed PSG data, while two (26, 28) provided actigraphy data. In the actigraphy subgroup, no significant difference was observed between the aMCI and NC groups [SMD = −0.00, 95%CI (−0.62~0.62), *P* = 1.00]. In the PSG subgroup, greater SE was observed in the NC group [SMD = −1.83, 95%CI (−2.99~-0.67), *P* = 0.002].

#### WASO

Seven (22–27, 29) included studies showed that there was no significant difference between the aMCI and NC groups [SMD = 0.18, 95%CI (−1.04~1.40), *P* = 0.77].

##### Subgroup analysis of WASO

Different measurements: five (22–25, 27) studies provided detailed PSG data, while two (26, 29) provided actigraphy data. In the actigraphy subgroup, no significant difference was observed between the aMCI and NC groups [SMD = −4.26, 95%CI (−13.31~4.80), *P* = 0.36]. In the PSG subgroup, WASO in the NC group was shorter than that in aMCI group [SMD = 1.29, 95%CI (0.48~2.11), *P* = 0.002].

#### Sleep Latency (SL)

Four (23, 26, 27, 29) included studies showed that there was no significant difference between the aMCI and NC groups [SMD = −0.02, 95%CI (−1.13~1.09), *P* = 0.97].

##### Subgroup analysis of SL

Different measurements: two (23, 27) studies provided detailed PSG data, while two (26, 29) provided actigraphy data. Both in the actigraphy subgroup and in the PSG subgroup, no significant difference was observed between the aMCI and NC groups [SMD = −0.71, 95%CI (−3.26~1.85), *P* = 0.59; SMD = 0.53, 95%CI (−0.19~1.26), *P* = 0.15; respectively].

#### SWS

Only one (27) included study provided SWS, and the parameter was detected by PSG. The result showed that the NC group had great SWS than the aMCI group [SMD = −3.52, 95%CI (−4.96~2.08), *P* < 0.00001].

#### Percent of Stage 1 of Non-rapid Eye Movement (N1%)

**Figure 5:**
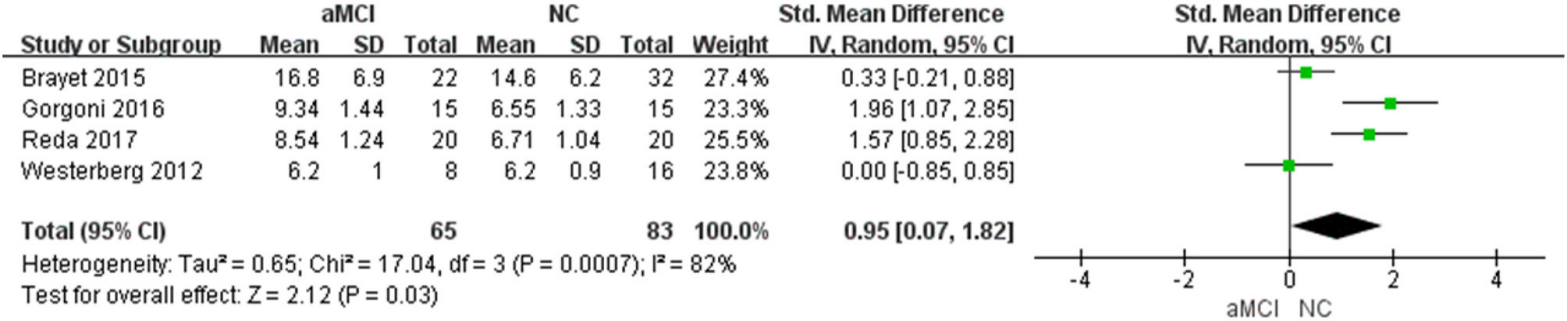
Meta-analysis of N1% between the aMCI and NC groups.

Four (22–24, 27) included studies showed that N1% in the aMCI group was greater than that in the NC group [SMD = 0.95, 95%CI (0.07~1.82), *P* = 0.03] (Figure 5).

#### Percent of Stage 2 of Non-rapid Eye Movement (N2%)

Four (22–24, 27) included studies showed that N2% in the NC group was greater than that in the aMCI group [SMD = −0.91, 95%CI (−1.26~0.57), *P* < 0.00001].

#### Percent of Slow Wave Sleep (SWS%)

Four (22–24, 27) included studies showed that there was no significant difference between the aMCI and NC groups [SMD = −2.05, 95%CI (−4.16~0.05), *P* = 0.06].

#### Percent of Rapid Eye Movement (REM%)

Four (22–24, 27) included studies showed that there was no significant difference between the aMCI and NC groups [SMD = −0.68, 95%CI (−1.80~0.45), *P* = 0.24].

#### Arousal Index (AI)

Two (23, 25) included studies showed that there was no significant difference between the aMCI and NC groups [SMD = −0.19, 95%CI (−0.59~0.22), *P* = 0.36].

#### Publication Bias

We used RevMan 5.3 to make the funnel plots. The figures appeared to have bilateral symmetry and we can conclude that no publication bias was evident.

## Discussion

### Main Findings and Discussions

In this study, we conducted a meta-analysis to clarify the differences of sleep structure as assessed by objective measures between aMCI patients and normal cognitive elderly people. We found that, SE of aMCI patients is significantly lower than that of normal cognitive elderly people, which is one of the most important indicators of sleep quality. Moreover, the SWS is significantly shorter in patients with aMCI compared with normal cognitive elderly people. However, compared with the normal control group, N1% in aMCI patients group increased, and N2% decreased. Nevertheless, there was no significant difference in TST, SL, AI, SWS%, REM%, and WASO between the two groups.

Previous studies have demonstrated that sleep disturbances and cognitive impairment have a bidirectional relationship (30, 31). Mounting epidemiologic evidence implicates disturbed sleep or lack of sleep as one of the risk factors for AD (8). Progressive worse changes in sleep quality, architecture, and neural regulation may constitute a contributing factor to cognitive decline (32). On the other hand, sleep disorders are extremely prevalent in neurodegenerative diseases. With cognitive impairment, there are further reductions of sleep quality, SWS, and percent REM, along with a profound disruption of circadian rhythmicity (32). Previous studies have confirmed that the characteristic sleep changes of AD patients are significantly decreased SE and reduced SWS (33, 34). In addition, studies have found that patients with MCI often suffer from sleep disorders. A review of 18 studies indicated that 14~59% of patients with MCI had sleep disturbances (35). However, generally speaking, epidemiologic studies used subjective sleep measures including questionnaire tools (such as PSQI and ESS) and self-reports (such as sleep diaries) to evaluate sleep quality (36). These subjective measures rely on the ability of patients and their caregivers to accurately recall sleep states (37). Indeed, subjective measures have poor concordance with objective measures and are subject to considerable recall limitations (38).

Hu et al. had conducted a meta-analysis of certain objective sleep parameter alterations in patients with MCI (39). But only one study providing data of an aMCI group was included. As one of the main subtypes of MCI, aMCI patients were our research objects in this study. Several parameters of sleep architecture were identified in our study. When compared with normal cognitive elderly controls, aMCI patients expressed lower SE and different sleep stage distribution including increased N1% and decreased SWS and N2%. Some epidemiological studies suggested that SWS is associated with better cognitive function, consistent with our results. A case-control study indicated that insufficiency of SWS may be the underlying mechanism of the association between insomnia and deficits in the executive control of attention (40). Memory impairment in aging is also indicated to be related to suppressed slow waves (41, 42). A trial demonstrated that enhancing slow wave activity conducted by acoustic stimulation was associated with improved morning word recall in individuals with aMCI (43). Accumulation of amyloid-β and tau aggregates, two histopathological markers of AD in the brain, are demonstrated to correlate with decreased SWS (44). What is more, a recent study suggests that slow wave loss could be associated with decreased cerebrospinal fluid flow, leading to lower protein aggregate clearance (45). However, SWS had significant differences while SWS% had none, the reason of which may be that the TST of patients with aMCI parallelly decreased, but without statistical significance.

The non-REM stage of aMCI patients had a distinctive change, with N1% increasing and N2% decreasing in aMCI patients. Targa A et al. found that some biomarkers changed with different sleep states in mild-moderate AD patients (46). For instance, there was a positive correlation between neurofilament light and N1%, and chitinase 3-like 1 was negatively correlated with N2%, SWS, and SE (46). Participants aged >65 years with cognitive impairment had significantly more N1 than those with no cognitive impairment, but the significance disappeared after adjustments for confounding variables (47). SE is another important index of sleep parameters. A cross-sectional study investigating the relationship between sleep and cognition in 2,932 elderly community members also found an association between low SE and MCI (48). Participants with SE <85% calculated using actigraphy showed a significantly decreased cognitive performance compared with those with SE ≥85% (49). With the decrease of SE, the accuracy of actigraphy decreased slightly (50). For mean WASO more than 30 min, actigraphy underestimated WASO compared to PSG (50). This possible mechanism may be that decreased SE is associated with decreased non-REM slow wave activity, leading to worse memory performance in those with amyloid deposition (51). In the future, improving SWS and SE to reduce the risk of memory loss or even AD is promising.

For the aMCI group in our study, not all sleep parameters were changed, and there was no significant difference in TST, SL, AI, REM%, and WASO. Previous studies have found that people with MCI have longer SL and shorter TST than healthy elderly people (39). In a 4-year longitudinal study of the relationship between cognitive aging and dementia in 2,238 healthy elderly people and 655 patients with MCI, increased SL could be an early clinical indicator of cognition decline in cognitively normal elderly people or patients with MCI (52). A large prospective study of sleep patterns measured by actigraphy reported that increased sleep fragmentation augmented the risk of AD and the rate of cognitive decline in older adults (53). Moreover, in the subgroup analysis, TST, SE, and WASO recorded by PSG between patients with aMCI and healthy participants were significantly different, while there was no difference in the actigraphy group. The different accuracy of objective sleep monitoring methods caused this distinction. PSG can provide information on the physiologic changes occurring in many different organ systems in relation to sleep stages and wakefulness. Actigraphy evaluates sleep state by recording human activities so that it is sensitive and reliable for predicting TST, SL, and SE. As compared with PSG, actigraphy is known to overestimate sleep and underestimate wake time (54).

### Strengths and Limitations

The strengths of this study mainly include the following three aspects. Firstly, aMCI patients are at a higher risk of developing AD, which deserves special attention. Secondly, the sleep parameters of the studies included in our study were measured by PSG and actigraphy, in order to avoid subjective assertion. Thirdly, our study summarized several case-control studies' outcomes to reach a more accurate agreement by the meta-analysis. Nevertheless, this study also has some limitations. Firstly, this study only searched literature in English, and there may be publication bias. Secondly, because the sleep data of objective measures are not easily obtained, the small sample size is also one of the limitations.

### Conclusion

Our study showed that patients with aMCI have significantly lower SE and shorter SWS, which are key points in elucidating the relationship between sleep disturbances and cognitive decline. Well-designed prospective studies and mechanism research are needed to provide more evidence in the future.

## Data Availability Statement

All datasets generated for this study are included in the article/supplementary material.

## Author Contributions

SR has primary responsibility for final content and is the study guarantor. SC, TL, and LZ together wrote this paper. TL and SC selected articles and extracted data. LZ, WT, and SR did study design and interpretation of results. LS, JL, WL, and GC helped with review selection and manuscript revision.

## Conflict of Interest

The authors declare that the research was conducted in the absence of any commercial or financial relationships that could be construed as a potential conflict of interest.
